# Native T1-mapping as a predictor of progressive renal function decline in chronic kidney disease patients

**DOI:** 10.1186/s12882-024-03559-1

**Published:** 2024-04-04

**Authors:** Zhaoyu Shi, Chen Sun, Fei Zhou, Jianlei Yuan, Minyue Chen, Xinyu Wang, Xinquan Wang, Yuan Zhang, Dmytro Pylypenko, Li Yuan

**Affiliations:** 1 Department of Nephrology, Affiliated Hospital of Nantong University, Nantong, 226000 Jiangsu China; 2 Nantong University Medical School, Nantong, Jiangsu China; 3 Department of Medical Imaging, Affiliated Hospital of Nantong University, Jiangsu, China; 4 GE Healthcare, MR Research China, Beijing, People’s Republic of China

**Keywords:** Chronic kidney disease, Renal fibrosis, Native T1 mapping, Magnetic resonance imaging

## Abstract

**Background:**

To investigate the potential of Native T1-mapping in predicting the prognosis of patients with chronic kidney disease (CKD).

**Methods:**

We enrolled 119 CKD patients as the study subjects and included 20 healthy volunteers as the control group, with follow-up extending until October 2022. Out of these patients, 63 underwent kidney biopsy measurements, and these patients were categorized into high (25–50%), low (< 25%), and no renal interstitial fibrosis (IF) (0%) groups. The study's endpoint event was the initiation of renal replacement therapy, kidney transplantation, or an increase of over 30% in serum creatinine levels. Cox regression analysis determined factors influencing unfavorable kidney outcomes. We employed Kaplan–Meier analysis to contrast kidney survival rates between the high and low T1 groups. Additionally, receiver-operating characteristic (ROC) curve analysis assessed the predictive accuracy of Native T1-mapping for kidney endpoint events.

**Results:**

T1 values across varying fibrosis degree groups showed statistical significance (F = 4.772, *P* < 0.05). Multivariate Cox regression pinpointed 24-h urine protein, cystatin C(CysC), hemoglobin(Hb), and T1 as factors tied to the emergence of kidney endpoint events. Kaplan–Meier survival analysis revealed a markedly higher likelihood of kidney endpoint events in the high T1 group compared to the low T1 value group (*P* < 0.001). The ROC curves for variables (CysC, T1, Hb) tied to kidney endpoint events demonstrated area under the curves(AUCs) of 0.83 (95%CI: 0.75–0.91) for CysC, 0.77 (95%CI: 0.68–0.86) for T1, and 0.73 (95%CI: 0.63–0.83) for Hb. Combining these variables elevated the AUC to 0.88 (95%CI: 0.81–0.94).

**Conclusion:**

Native T1-mapping holds promise in facilitating more precise and earlier detection of CKD patients most at risk for end-stage renal disease.

## Background

Chronic kidney disease (CKD) is increasingly recognized as a global public health problem [1]. However, predicting the evolution of CKD remains challenging. This progressive deterioration places a significant burden on patients and their families, not to mention the economic strain on society. Consequently, the early identification of CKD patients with rapidly deteriorating renal functions, coupled with timely interventions and treatments, is vital for improving kidney health or delaying CKD progression. Kidney fibrosis, characterized by excessive deposition of extracellular matrix, is a key driver of CKD progression [2]. At present, the histopathological evaluation of renal tissue obtained through percutaneous renal biopsy is the gold standard for assessing the degree of renal fibrosis. However, renal biopsy is invasive and faces limitations due to the nature of the samples collected, potentially leading to biased evaluations of the overall kidney fibrosis. Furthermore, patients often have a low tolerance for repeated renal biopsies. Thus, there is a pressing need for a non-invasive, repeatable method.

Magnetic resonance imaging (MRI) has undergone rapid advancement over recent decades. Numerous studies have demonstrated that functional and molecular MRI techniques provide non-invasive, valuable tools for assessing interstitial fibrosis and predicting prognosis in kidney diseases [3]. Native T1-mapping is a novel non-enhanced quantitative MRI technique that gauges the extent of tissue fibrosis by directly measuring the tissue's T1 values. Currently, Native T1 mapping has been utilized to assess cardiac and hepatic fibrosis [4, 5], suggesting its potential as an alternative tool for non-invasive evaluations of renal diseases [6]. In a prior study, we discovered that Native T1 mapping might offer strong diagnostic capabilities in assessing renal function and in the non-invasive detection of chronic glomerulonephritis fibrosis [7].

To the best of our knowledge, ours is the first study exploring the correlation between native T1 mapping and the prognosis of CKD patients. The primary objective of this research was to determine if the renal T1 value can forecast a progressive decline in renal function among a group of CKD patients.

## Methods

### Study design and subject recruitment

This research project received approval from the Ethics Committee of the Affiliated Hospital of Nantong University (2019-K070). Written informed consent was obtained from all participants.

From September 2019 to October 2021, adults aged between 18 and 70 years diagnosed with CKD groups 1 to 4 (G1-4) were consecutively enrolled in our study. The definition and staging criteria for CKD follow the Kidney Disease Outcome Quality Initiative(K/DOQI) guidelines [8]. Participants were excluded based on the following criteria: 1) Contraindications for MRI examination, such as the presence of metal objects in the body or an inability to cooperate with the examination; 2) Detection of renal abnormalities during the MRI examination, including large renal cysts, solitary kidneys, hydronephrosis, tumors, or other renal anomalies; 3) Poor image quality.

After applying these criteria, 119 patients remained in our study groups. Additionally, 20 healthy controls without kidney disease were recruited. However, one individual was excluded due to the detection of proteinuria through routine urinalysis.

All MRIs performed in the same facility within one week before kidney biopsy. All subjects underwent MRI after fasting and refraining from drinking for at least 6 h. All of them did not have major cardiovascular diseases such as heart failure or myocardial infraction.

Patients were followed up every 12 months until October 31, 2022. The study's endpoint was defined as the initiation of renal replacement therapy, receipt of a kidney transplant, or an increase in baseline serum creatinine (SCr) by more than 30%.

**Fig. 1:**
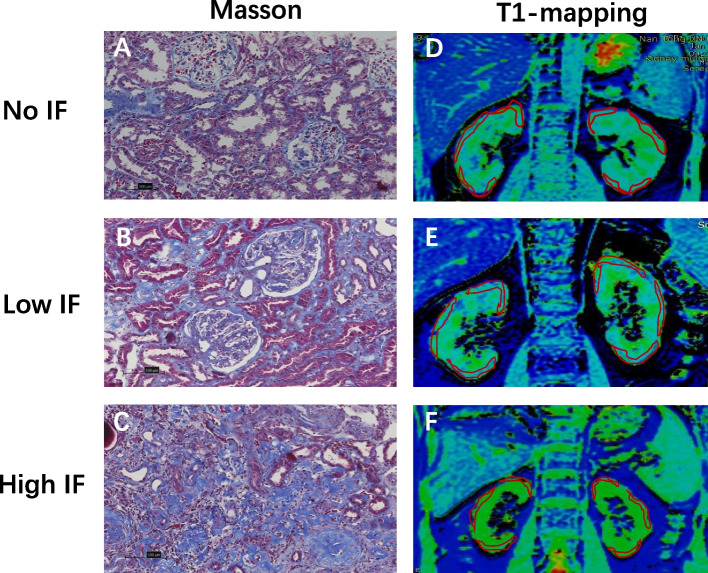
**a**-**c** Masson-stained section reveals progressive tubulointerstitial fibrosis, as no, low, and high IF, respectively (20 ×). **d**-**f** The T1-mappings of varying degrees of fibrosis(D: T1 = 1622ms; E: T1 = 1796ms; F: T1 = 2056ms)

Renal biopsies were conducted on 63 patients. The criteria for the kidney biopsy: 1. glomerular hematuria with any degree of proteinuria; 2. isolated proteinuria > 1 g/day;3. unexplained renal disease. Formalin-fixed renal tissues were embedded in paraffin. Two-micrometre-thick paraffin sections were prepared and stained with Masson's trichrome. The degree of pathological injury was scored using the Katafuchi semiquantitative scoring system [9]. The details of the pathology scores are shown in Table 1. According to the renal pathology results, the degree of renal interstitial fibrosis (IF) in this study was graded from 0 to 50%. Consequently, renal IF was categorized into high (25–50%), low (< 25%), and no IF groups (0%) as depicted in Fig. 1.

**Table 1:** A semi-quantitative standard for calculating the scores of glomerular, tubular interstitial and vascular lesions

Scores	Glomerular lesion score			Tubulointerstitial lesion score			Vascular lesion score	
	Glomerular cell proliferation (%)	Segmental lesions (%)	Glomerular sclerosis (%)	Interstitial fibrosis (%)	Tubular atrophy (%)	Interstitial inflammatory cell infiltration (%)	Vascular thickening (%)	Hyaline degeneration (%)
0	NA	0	0	0	0	0	0	0
1	≤ 25	≤ 10	≤ 10	≤ 25	≤ 25	≤ 25	≤ 25	≤ 25
2	25–50	10–25	10–25	25–50	25–50	25–50	25–50	25–50
3	50–75	25–50	25–50	≥ 50	≥ 50	≥ 50	≥ 50	≥ 50
4	≥ 75	≥ 50	≥ 50	NA	NA	NA	NA	NA

*NA* Not applicable

### Clinical parameters

The following parameters were collected for each participant: age, gender, presence or absence of hypertension and diabetes, body mass index (BMI), 24-h urine protein (24 h-UP), albumin (Alb), hemoglobin (Hb), cystatin C (CysC), SCr, and estimated glomerular filtration rate (eGFR) calculated using the Chronic Kidney Disease Epidemiology Collaboration (CKD-EPI) formula [10].

### Magnetic resonance imaging

MRI examinations were conducted for each subject in the coronal view by GE 3.0 T magnetic resonance scanner(Discovery MR750, GE Healthcare, Milwankee, WI). The scan parameters applied were: slice thickness = 5 mm, spacing = 1 mm, number of slices = 10, field of view ranging from 30 cm × 30 cm to 36 cm × 36 cm, matrix = 192 × 128, number of excitations = 1, and acceleration factor = 2. A respiration trigger was also utilized. The scan duration was 3 min. Using the vendor-provided post-processing software embedded in a GE advanced workstation (ADW4.6), coronal renal T1 maps were generated for each subject [7]. On the renal T1 maps, three regions of interest (ROIs) were manually delineated on the upper, middle, and lower sections of each renal cortex by a senior radiologist with 15 years of experience (Fig. 1).

### Statistical analysis

The data were analyzed using SPSS 25.0. Metric data with a normal distribution were represented as mean ± standard deviation, and one-way analysis of variance was used for comparisons between multiple groups. Metric data with a non-normal distribution were represented as M (Qr), and the Wilcoxon rank-sum test was used for comparisons between two or more groups. Qualitative data were expressed as a percentage. The Spearman correlation coefficient was employed to evaluate the relationship between T1 values and pathological scores. Kaplan–Meier survival curves were used to analyze the probability of no kidney endpoint events occurring between the high and low T1 groups over time. Cox regression analysis was used to explore the association between T1 mapping, clinical indices, and the occurrence of kidney endpoint events. Receiver operating characteristic (ROC) curves were used to evaluate the accuracy of different variables in predicting renal endpoint events. A *p*-value < 0.05 was considered statistically significant in all analyses.

## Results

The baseline characteristics of the 119 patients with CKD G1-4 and the specific pathological types of 63 patients can be found in Table 2. The study cohort included 41 patients with CKD G1, 33 with CKD G2, 30 with CKD G3, and 15 with CKD G4. On average, patients were followed up for a duration of 21.0 ± 8.7 months.

**Table 2:** The demographics and Clinical parameters of CKD sub-groups

	CKD G1(*n* = 41)	CKD G2(*n* = 33)	CKD G3(*n* = 30)	CKD G4(*n* = 15)	F/χ^2^	*P*
Age(years)	42 ± 14	50 ± 13	50 ± 12	47 ± 11	3.771	0.013
Gender(% Male)	63	64	60	53	0.186	0.905
eGFR (ml/min/1.73m^2^)	113 ± 21	75 ± 9	47 ± 8	22 ± 4	211.6	< 0.001
Hypertension (%)	27	61	57	87	7.036	< 0.001
24-h urinary protein(g)	3.1 (2.1,4.1)	4.6 (2.9,6.3)	3.4 (2.3,4.5)	5.9 (2.9,8.9)	2.446	0.068
Albumin(g/L)	31 ± 8	30 ± 10	34 ± 7	31 ± 6	1.467	0.227
Haemoglobin (g/L)	133 ± 12	137 ± 23	122 ± 20	112 ± 20	8.337	< 0.001
Body mass index (kg/m^2^)	25 ± 4	25 ± 4	24 ± 4	25 ± 4	0.400	0.753
Cystatin C(mg/L)	0.8 ± 0.2	1.1 ± 0.2	1.4 ± 0.3	2.4 ± 0.5	125.7	< 0.001
Diabetes(%)	12	42	37	40	3.484	0.018
Serum creatinine (μmol/L)	68 ± 14	94 ± 17	136 ± 30	294 ± 89	144.7	< 0.001
Diuretics(%)	24	31	17	13	0.994	0.398
Number of renal biopsy	28	19	14	2		
IgAN	12	3	6	0		
MN	8	5	0	0		
MCD	3	2	1	0		
FSGS	2	1	1	0		
DKD	0	5	3	2		
Others	3	3	3	0		

*IgAN* IgA nephropathy, *MN* Membranous glomerulonephritis, *MCD* Minimal change disease, *FSGS* Focal segmental glomerulosclerosis, *DKD* Diabetic kidney disease

### Data analysis between T1 values and pathological findings

**Fig. 2:**
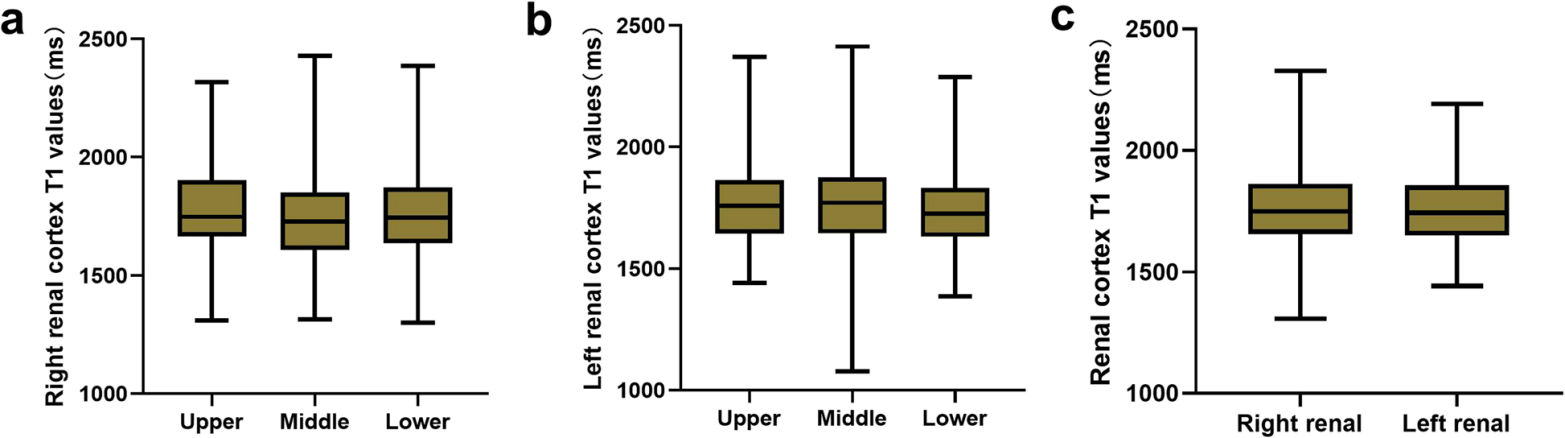
T1 comparison between each sub-regions of right kidney (**a**) and left kidney (**b**), and between bilateral kidneys (**c**). No significant T1 difference of the renal cortex was found (all *p* > 0.05)

**Fig. 3:**
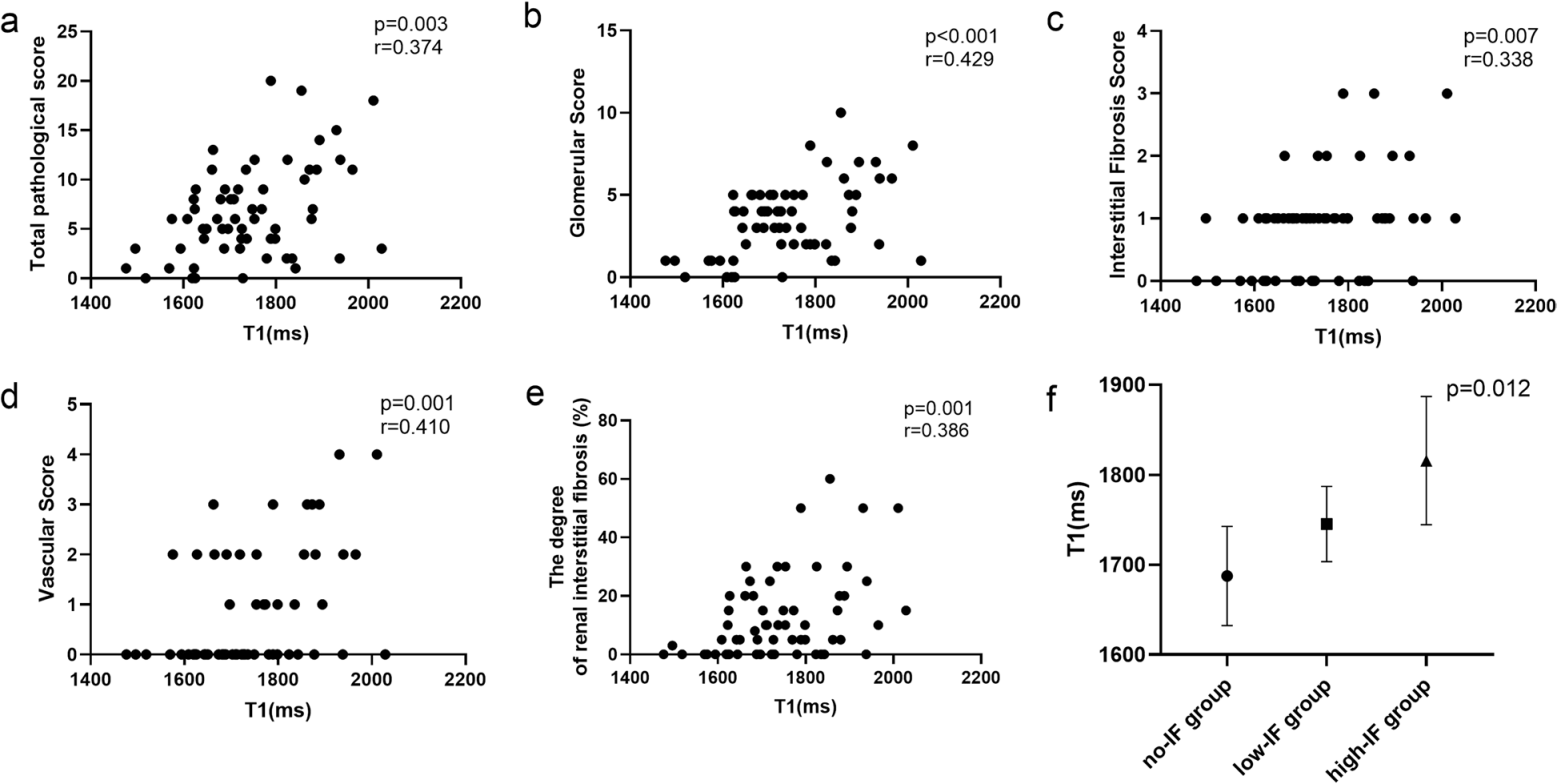
**a-e** The cortical T1 value of the kidney was positively correlated with the biopsy pathological scores. **f** There was a statistically significant difference in T1 values between different levels of fibrosis (F = 4.772, *p* < 0.05)

There was no significant difference in T1 values within different regions of the same kidney or between the two kidneys (all *p* > 0.05) (Fig. 2). The final T1 value for a kidney was calculated as the average of its T1 values. The cortical T1 value of the kidney positively correlated with the total pathological score (*r* = 0.374, *p* = 0.003), glomerular score (*r* = 0.429, *p* < 0.001), fibrosis score (*r* = 0.338, *p* = 0.007), vascular score (*r* = 0.410, *p* = 0.001), and the percentage of fibrosis in the kidney (*r* = 0.386, *p* = 0.001). There was a statistically significant difference in T1 values across different levels of fibrosis (F = 4.772, *p* < 0.05) (Fig. 3).

### T1 Analysis for the control group and the study groups

**Fig. 4:**
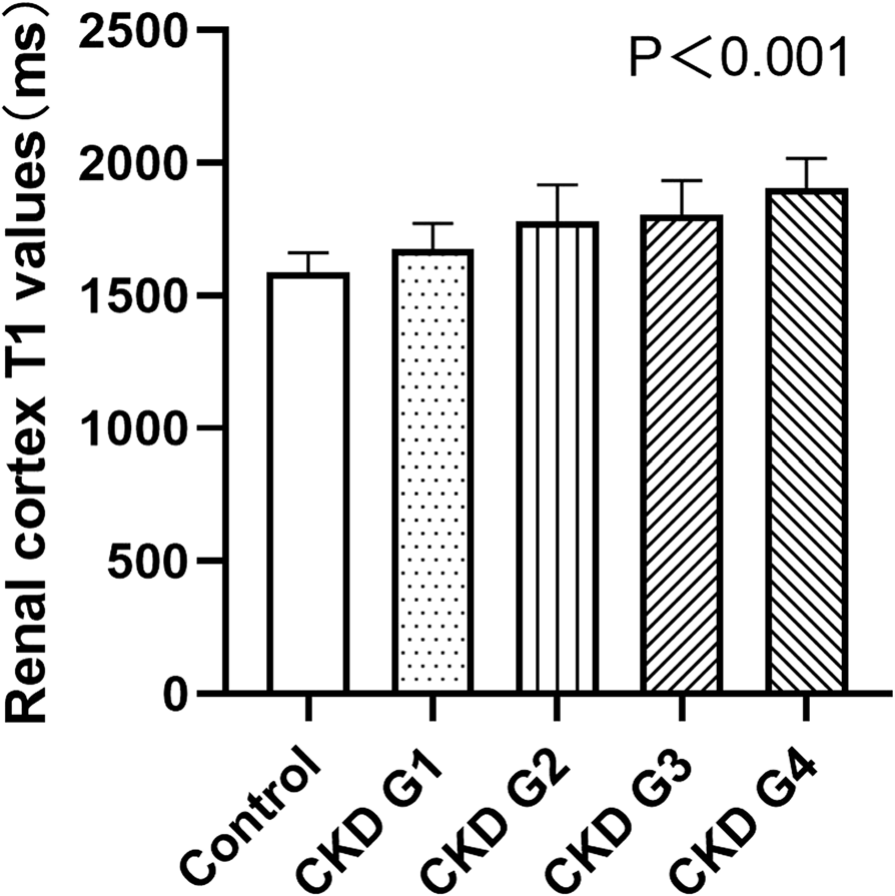
Compare the T1 values in patients with different stages of CKD in the control group and the study groups

Compared to the control group's T1 values, there were statistically significant differences in T1 values among patients with various stages of CKD. The T1 values were as follows: control group (1595 ± 78) ms, CKD G1 (1673 ± 97) ms, CKD G2 (1781 ± 136) ms, CKD G3 (1803 ± 131) ms, and CKD G4 (1905 ± 114) ms (F = 22.190, *P* < 0.001). In post-hoc tests, T1 values between any two groups were statistically significant (*p* < 0.05), except for between stages 2 and 3 (*p* > 0.05). ( Fig. 4).

### Diagnostic performance for predicting renal outcome

The follow-up period for the study ranged from 12 to 38 months, with an average duration of 21.0 ± 8.7 months. By the end of the follow-up, 45 patients from the study group had reached the endpoint: 14 patients underwent dialysis, 4 received kidney transplants, and 27 experienced a Scr increase of more than 30%. All the patients with diabetes (10) had reached the endpoint, the remains were the patients with glomerular diseases (35). No patients from the control group reached the endpoint.

**Fig. 5:**
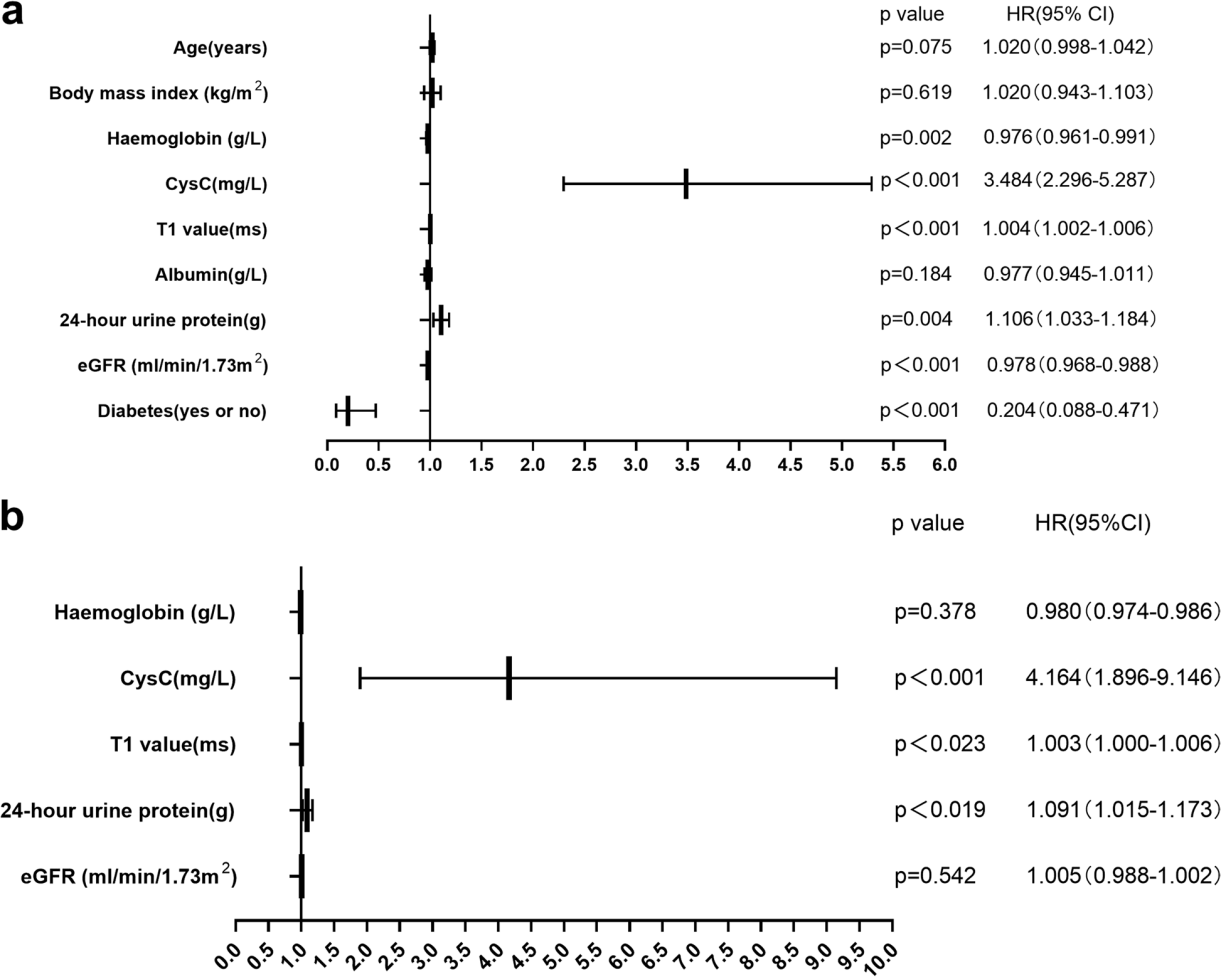
**a** Univariate analysis showed that diabetes, eGFR, 24-h urine protein, T1 value, CysC, and Hb were all associated with endpoint events (*P* < 0.05). **b** Multivariate analysis (fully adjusted) indicated that 24-h urine protein, CysC, Hb, and T1 value were associated with endpoint events(*P* < 0.05). Endpoint events: the initiation of renal replacement therapy, receipt of a kidney transplant, or an increase in baseline serum creatinine by more than 30%

Cox regression analysis was conducted with the occurrence of endpoint events as the dependent variable. The univariate analysis revealed associations between endpoint events and factors such as diabetes, eGFR, 24-h urine protein, T1 value, CysC, and Hb (*P* < 0.05). A fully adjusted multivariate analysis indicated associations between endpoint events and 24-h urine protein, CysC, Hb, and T1 value (*P* < 0.05) (Fig. 5).

**Fig. 6:**
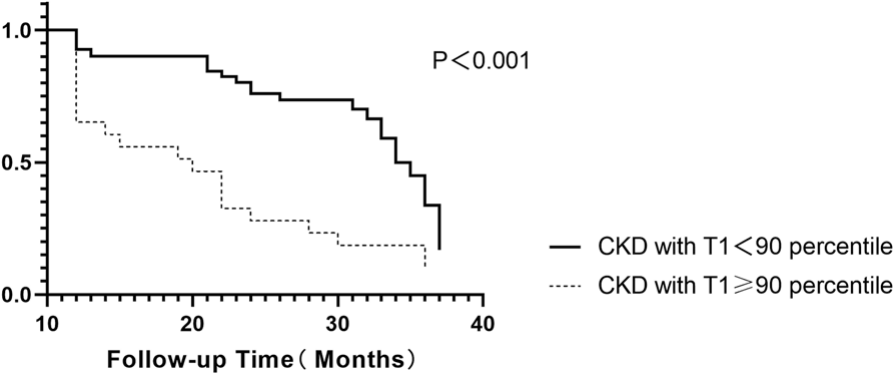
Kaplan–Meier analysis showed statistically significant differences (*P* < 0.001) between the high(patients with T1 values exceeding 90% of the highest value in the cohort) and low T1 groups(patients with T1 values below 90% of the highest value in the cohort) in terms of prognosis over time (months)

Patients with T1 values exceeding 90% of the highest value in the cohort were classified into the high T1 group, while those with values below 90% of the highest value in the cohort were categorized into the low T1 group. The high T1 group consisted of 23 CKD patients, 19 of whom experienced renal endpoint events during the follow-up. In contrast, out of the 96 patients in the low T1 group, 26 encountered endpoint events. Therefore, the likelihood of encountering endpoint events in the high T1 group was threefold compared to the low T1 group. Kaplan–Meier analysis revealed a statistically significant difference in prognosis between the high and low T1 groups in terms of the occurrence of kidney endpoint events over time (months) (*P* < 0.001) (Fig. 6).

**Fig. 7:**
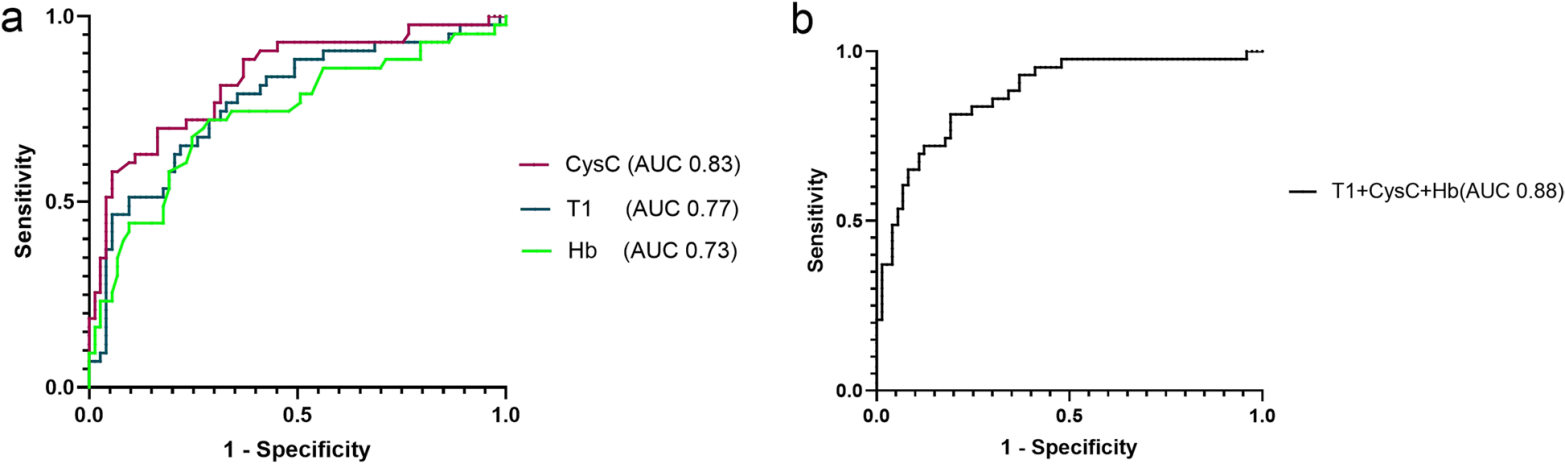
**a** Receiver operating characteristic (ROC) curve and area under the curve (AUC) of CysC(in red), T1 (in blue), and Hb (in green) for their prediction of renal endpoint events in CKD patients. **b** ROC curve and AUC of the 3 variables combined

The ROC curves, which correspond to variables linked with renal endpoint events (CysC, T1, and Hb), are depicted in the subsequent figure. The area under the curve(AUC) for CysC stood at 0.83 (95% CI: 0.75–0.91), T1 at 0.77 (95% CI: 0.68–0.86), and Hb at 0.73 (95% CI: 0.63–0.83). The combined ROC curve of CysC, T1, and Hb demonstrated an enhanced AUC of 0.88 (95% CI: 0.81–0.94) (Fig. 7).

## Discussion

Our study results indicate a correlation between T1 values and various pathological scores, as well as the occurrence of adverse renal events. Elevated cortical T1 values in CKD patients correlate with a poorer prognosis and an increased likelihood of adverse renal events. This suggests that native T1 mapping might serve as a noninvasive biomarker for evaluating fibrosis and predicting prognosis in CKD patients.

While studies have highlighted the potential of native T1 mapping to assess CKD patients in kidney disease research, findings have consistently shown that T1 values in CKD patients are significantly longer than those in control groups [11]. Our prior study with chronic glomerulonephritis patients yielded similar results [7]. Additionally, native T1 mapping has been suggested to have a pivotal role in evaluating renal function impairment in transplant recipients and IgA nephropathy patients [12, 13]. In our current research, we found notable differences in kidney T1 values between healthy control groups and CKD G1-4 patients, further cementing the strong relationship between kidney T1 values and renal function.

CKD covers a spectrum of etiologies, advancing through shared pathological mechanisms such as glomerular capillary hypertension and hyperfiltration, inflammation, vascular rarefaction, hypoxia, and fibrosis [14, 15]. Increasing evidence suggests MRI techniques hold immense promise in evaluating and quantifying kidney disease's pathophysiological processes. Functional MRI can assess renal tissue perfusion, oxygenation, interstitial diffusion, cellular metabolism, and molecular expression with techniques like diffusion weighted imaging(DWI), diffusion tensor imaging(DTI), blood oxygen level development(BOLD), and magnetic resonance elastography(MRE) [16–19]. Additionally, recent research underscores the potential of T1 mapping in assessing renal fibrosis. Preliminary research has shown that T1 mapping effectively gauges renal fibrosis in mice with CKD [20]. Friedli et al. [21] discovered that T1 values correlate well with fibrosis and inflammation, offering an evaluation metric for transplanted kidneys' interstitial fibrosis. Graham-Brown et al. [13] reported rising T1 values in patients with high interstitial damage scores. Our biopsy analysis of 63 patients revealed that T1 values align with fibrosis scores, reflecting fibrosis levels. Our preceding study also corroborated these findings. Nevertheless, more extensive multicenter studies are essential to validate T1 mapping. Currently, there's a dearth of longitudinal studies that delve into functional MRI's predictive value in assessing renal function alterations and prognosis. Some studies using BOLD to assess renal oxygenation have shown correlations with declining renal function, validating the chronic hypoxia hypothesis, and paving the way for future research [22].

In our study involving 119 CKD patients, we determined that T1 value might be an independent risk factor for CKD prognosis. Cox regression analysis, focusing on adverse kidney events, revealed correlations with age, diabetes, baseline eGFR, 24-h urinary protein, T1 value, CysC, Hb, and endpoint events. Subsequent multivariate analysis suggested that, after accounting for confounding factors, 24-h urine protein, CysC, Hb, and T1 were independent risk factors. Past research has shown anemia accelerates kidney function decline and ups end stage renal diseases incidence rates [23, 24]. 24-h urine protein is also a significant risk factor for CKD onset [25], while CysC indicates kidney filtration function and can flag early declines in kidney function [26]. Our ROC curve analysis determined that CysC, with an AUC value of 0.83, was the strongest determinant for adverse kidney events, followed by T1 (AUC = 0.77) and Hb (AUC = 0.73). But combining these indicators pushed the AUC value to 0.88, amplifying the predictive capability for adverse kidney events.

We would like to observe whether there is a specific group, within which the prognosis of the kidneys is worse. In our study, T1 values ranged from 1600 to 2000. We attempted to group patients reasonably for the first time. Eventually, patients with T1 values exceeding 90% of the highest value in the cohort were classified into the high T1 group, while those with values below 90% of the highest value in the cohort were categorized into the low T1 group. Grouping T1 values showed that the high T1 group had over thrice the likelihood of adverse events than the low T1 group. With the most extended follow-up spanning 38 months, the two CKD groups showed significant differences over time in terms of the absence of adverse kidney events, boosting T1 mapping's potential to predict CKD prognosis.

This study, however, had some limitations. First, it's a single-center, retrospective cohort study. Even after accounting for multiple factors, confounding variables persist, with potential confounders overlooked due to the retrospective nature. Second, no patients who underwent renal biopsy had more than 50% renal IF. Third, this study only focused on CKD; more research is required to investigate the clinical value of T1 mapping in other renal diseases. Last but not least, researches on the utility of T1 mapping compared to other modalities and its correlation with markers of tubular injury are still lacking. Future research will expand the cohort, add more markers and redo MRI scans on the previous subjects to affirm the prognostic value of T1 mapping for CKD patients.

In conclusion, native T1-mapping not only evaluates the kidney function of CKD patients but also mirrors their prognosis. It holds promise as a novel non-invasive method for predicting adverse kidney events in CKD patients.

## Conclusion

Native T1-mapping has the potential to significantly improve the identification of CKD patients with a higher risk of progressing to end-stage renal disease by providing an early and accurate assessment of renal fibrosis and dysfunction.

## Data Availability

The datasets used and analysed during the current study are available from the corresponding author on reasonable request.

## Abbreviations

CKD: Chronic kidney disease
IF: Interstitial fibrosis
ROC: Receiver operating characteristic
CysC: Cystatin C
Hb: Hemoglobin
AUC: Area under the curve
MRI: Magnetic resonance imaging
K/DOQI: Kidney disease outcome quality initiative
SCr: Serum creatinine
BMI: Body mass index
24 h-UP: 24-Hour urine protein
Alb: Albumin
eGFR: Estimated glomerular filtration rate
CKD-EPI: Chronic Kidney Disease Epidemiology Collaboration
ROI: Regions of interest
DWI: Diffusion weighted imaging
DTI: Diffusion tensor imaging
BOLD: Blood oxygen level development
MRE: Magnetic resonance elastography
